# Optimal dose determination of enerisant (TS-091) for patients with narcolepsy: two randomized, double-blind, placebo-controlled trials

**DOI:** 10.1186/s12888-022-03785-7

**Published:** 2022-02-22

**Authors:** Yuichi Inoue, Makoto Uchiyama, Hideo Umeuchi, Koichi Onishi, Hiroki Ogo, Iwao Kitajima, Isao Matsushita, Izumi Nishino, Naohisa Uchimura

**Affiliations:** 1 Japan Somnology Center, Institute of Neuropsychiatry, 5-10-10 Yoyogi, Shibuya-ku, Tokyo 151-0053 Japan; 2 Department of Somnology, Tokyo Medical University, 6-7-1 Nishishinjuku, Shinjuku-ku, Tokyo 160-0023 Japan; 3 Department of Psychiatry, Nihon University School of Medicine, 30-1 Oyaguchi-kamicho, Itabashi, , Tokyo 173-8610 Japan; 4 Tokyoadachi Hospital, 5-23-20 Hokima, Adachi, Tokyo 121-0064 Japan; 5 Development Headquarters, Taisho Pharmaceutical Co., Ltd., 3-24-1 Takada, Toshima-Ku, Tokyo 170-8633 Japan; 6 Department of Neuropsychiatry, Kurume University School of Medicine, 67 Asahi-machi, Kurume, Fukuoka, 830-0011 Japan

**Keywords:** Enerisant, TS-091, Narcolepsy, Histamine, Clinical trial, MWT, ESS

## Abstract

**Background:**

The histamine H3 receptor has emerged as one of the most promising targets of novel pharmacotherapy for narcolepsy. Studies now aim to investigate the optimal dose of enerisant, a novel H3 antagonist/inverse agonist, for the treatment of excessive daytime sleepiness in patients with narcolepsy.

**Methods:**

We conducted two phase 2, fixed-dose, double-blind, randomized, placebo-controlled trials in patients with narcolepsy. The first phase 2 study (Study 1) was conducted to investigate the efficacy and safety of enerisant at dosages of 25, 50, and 100 mg/day administered for 3 weeks based on the results of a phase 1 study conducted on healthy volunteers. The primary endpoint was mean sleep latency in maintenance of wakefulness test (MWT), and the secondary endpoint was the total score on the Epworth Sleepiness Scale (ESS). The dosages of enerisant in the second phase 2 study (Study 2) were set at 5 and 10 mg/day based on the simulation of receptor occupancy results from positron emission tomography study.

**Results:**

Forty-six and fifty-three patients were randomized in Study 1 and Study 2, respectively. The efficacy of enerisant was partially confirmed in Study 1 with ESS; however, the doses were not tolerated, and there were many withdrawals due to adverse events (mainly insomnia, headache, and nausea). The doses in Study 2 were well tolerated, with a lower incidence of adverse events in Study 2 than in Study 1, although the efficacy could not be confirmed with MWT and ESS in Study 2.

**Conclusions:**

The optimal dose of enerisant could not be determined in these two studies. Although enerisant has a favorable pharmacokinetic profile, it is thought to have large interindividual variabilities in terms of efficacy and safety, suggesting the necessity of tailored dosage adjustments.

**Trial registration:**

ClinicalTrials.gov identifier: NCT03267303; Registered 30 August 2017 (Study 2).

Japic identifier: JapicCTI-142529; Registered 7 May 2014 (Study 1) and JapicCTI-173689; Registered 30 August 2017, https://www.clinicaltrials.jp/cti-user/trial/ShowDirect.jsp?clinicalTrialId=29277 (Study 2).

**Supplementary Information:**

The online version contains supplementary material available at 10.1186/s12888-022-03785-7.

## Background

Narcolepsy is a sleep disorder characterized by a pathologically increased propensity for both sleep onset and rapid eye movement sleep, causing impairment of work performance and quality of life [1], as well as sleepiness-related driving accidents. Currently, several drugs, including modafinil/armodafinil, methylphenidate, pemoline, and sodium oxybate, are available for the treatment of narcolepsy. However, these drugs have drawbacks, such as abuse potential, problems in cardiac safety, tolerability, and adherence issues; furthermore, some patients are refractory to these drugs [2]. Therefore, new pharmacotherapies with different mechanisms and improved safety are desirable.

The histamine H3 receptor has emerged as one of the most promising targets of novel pharmacotherapy for narcolepsy. Histaminergic neurons are located downstream of the orexin system, the dysfunction of which is involved in the mechanism of narcolepsy, and the histamine H3 receptor in the central nervous system has a critical role in the regulation of sleep–wake cycles by modulating histaminergic tones as an autoreceptor [3]. To date, many histamine H3 receptor antagonists/inverse agonists with diverse scaffolds have been synthesized, and some have been tested in clinical trials [4]. Among these, pitolisant was approved for the treatment of excessive daytime sleepiness (EDS) or cataplexy associated with narcolepsy in 2016 in Europe and in 2019 in the United States [5]. There have been no reports on the dose–response relationship of pitolisant for the treatment of EDS or cataplexy in patients with narcolepsy. The drug is approved for a wide range of doses, from 4.5 to 36 mg/day, with recommendations of patient-specific dose adjustments based on efficacy, safety, and drug–drug interaction (DDI) risks. Thus, the appropriate dose of histamine H3 receptor antagonists/inverse agonists to exert wake-promoting effects and the relationship between histamine H3 receptor occupancy and efficacy for the treatment of narcolepsy has not been fully established.

Enerisant, [1-(4-{3-[(2R)-2-methylpyrrolidin-1-yl] propoxy} phenyl)-1H-pyrazol-4-yl] (morpholin-4-yl) methanone, is an antagonist/inverse agonist of histamine H3 receptor synthesized by Taisho Pharmaceutical Co., Ltd (Tokyo, Japan) [6]. In vitro studies have shown that enerisant is a potent, highly selective, and competitive antagonist for the histamine H3 receptor, with a more than 3,000-fold selectivity over other histamine receptor subtypes [6]. Notably, enerisant does not have any affinity for σ1 receptors, unlike pitolisant [7]. Enerisant is rarely metabolized in humans, and up to 90% of the dose is excreted as an unchanged form through urine by 48 h after administration. These findings suggest that DDI do not occur even when enerisant is used in combination with cytochrome P450 (CYP) inhibitors or inducers, unlike other histamine H3 receptor antagonists [8]. Therefore, it is possible that enerisant has superior pharmacokinetic (PK) profiles than pitolisant. In addition, because of higher receptor selectivity and superior PK profiles, enerisant can be a suitable tool to determine the appropriate dosage of histamine H3 receptor antagonists/inverse agonists for the treatment of narcolepsy. Enerisant demonstrated tolerability at doses of 50 and 100 mg for 7 days in a multiple ascending dose (MAD) study of Japanese healthy individuals. In a MAD study conducted on healthy individuals in the United States, tolerability up to 150 mg for 10 days was confirmed [Unpublished data].

The aim of this report was to investigate the optimal dose of enerisant for the treatment of EDS in patients with narcolepsy. In the 1st phase 2 study (Study 1) for patients with narcolepsy, the dose was decided based on the result of a phase 1 study and nonclinical studies, and in the 2nd phase 2 study (Study 2), the dose was chosen according to the results of the histamine H3 receptor occupancy study using positron emission tomography (PET) ligand on healthy subjects [9].

## Methods

### Study design

#### Study 1

**Fig. 1:**
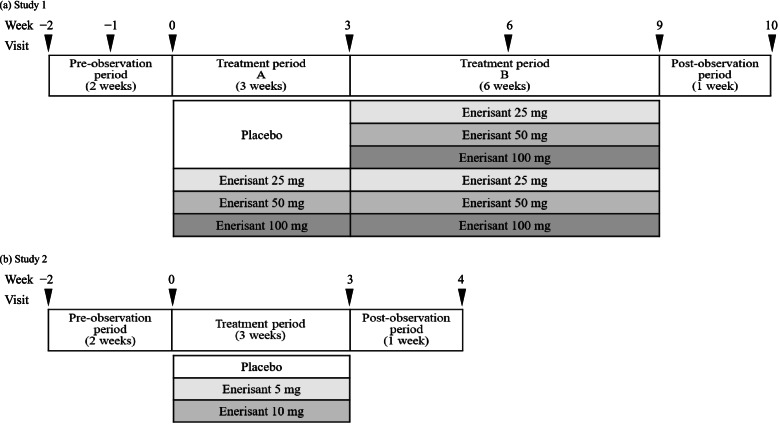
Study design for Study 1 (**a**) and Study 2 (**b**)

This multicenter, randomized, placebo-controlled, double-blind, parallel-group comparative phase 2 study was conducted at 19 sites in Japan (Fig. 1a). Study 1 consisted of four consecutive periods: a preobservation period (2 weeks), treatment period A (3 weeks), treatment period B (6 weeks), and a postobservation period (1 week). Treatment period A was designed to evaluate the safety and efficacy of enerisant in patients with narcolepsy, whereas treatment period B was designed mainly to evaluate the safety of enerisant. The target sample size of each group (enerisant 25, 50, 100 mg, and placebo groups) was set at 12, 12, 9, and 12 participants, respectively, for a total of 45 participants, considering that narcolepsy is a rare disease and this was an exploratory study.

#### Study 2

Study 2 was designed after Study 1 was completed. This multicenter, randomized, placebo-controlled, double-blind, parallel-group comparative phase 2 study was conducted at 19 sites in Japan and 4 sites in Korea (Fig. 1b). Study 2 consisted of three consecutive periods: a preobservation period (2 weeks), a treatment period (3 weeks), and a postobservation period (1 week). The treatment period was designed to evaluate the safety and efficacy of enerisant in patients with narcolepsy. The target sample size was set at 16 participants/group with a total of 48 participants, considering the feasibility in patients with the rare disease and the exploratory evaluations of efficacy and safety as the study objective.

### Patients

#### Study 1

Eligible patients, aged 16–64 years, had received a diagnosis of narcolepsy with/without cataplexy based on the second edition of the International Classification of Sleep Disorders (ICSD-2) [10]. Patients who reported daytime dozing off for at least 4 days and 7 times within 7 days, calculated from their sleep diaries in the preobservation periods, were considered eligible. Exclusion criteria were patients with an apnea hypopnea index of ≥ 15, periodic limb movement with an arousal index of ≥ 10 on baseline overnight polysomnography (PSG), sleep latency (SL) of ≥ 20 min at any session, or mean SL of ≥ 11 min at 4 sessions on baseline MWT. These criteria items were set with reference to the criteria of the phase 3 study on the use of modafinil in patients with narcolepsy that was previously conducted in Japan, or other relevant studies on pitolisant and JZP-110 (NCT01638403) [11–13]. Patients with complications of organic brain diseases, epilepsy, obstructive respiratory diseases, sleep disorders other than narcolepsy, or significant cardiovascular disease were also excluded. Throughout the study period, the use of drugs that may affect the efficacy and safety assessment of enerisant, such as central nervous system stimulants, anxiolytics, most antidepressants (only clomipramine was allowed to be used at a fixed dose during the study period if the drug was used for cataplexy prior to enrollment in the study), and antihistamines, was prohibited. Only 5 participants used clomipramine during the study (2, 2, 0, and 1 subject in the 25 mg, 50 mg, 100 mg, and placebo groups, respectively).

#### Study 2

Eligible patients were aged 16–64 years at the time of informed consent, with a confirmed diagnosis of narcolepsy type 1 or type 2 based on the third edition of the International Classification of Sleep Disorders (ICSD-3) [14]. Patients with symptoms of EDS occurring at least 4 days and 7 times within 7 days in the preobservation periods were eligible for participation. To enroll patients with a milder severity of narcolepsy, the exclusion criteria on MWT were changed from Study 1; patients with mean SL = 0 min at any session or ≥ 20 min in at least two of the four sessions on baseline MWT were excluded. Other exclusion criteria were patients with an apnea hypopnea index of ≥ 15, periodic limb movement associated arousal index of ≥ 10, or total sleep time of ≤ 360 min on baseline PSG. Patients with complications of organic brain diseases, epilepsy, obstructive respiratory diseases, sleep disorders other than narcolepsy, and significant cardiovascular disease were also excluded. Throughout the study period, the use of drugs that may affect the efficacy and safety assessment of enerisant, such as central nervous system stimulants, anxiolytics, antidepressants (including clomipramine), and antihistamines, was prohibited.

### Treatments

#### Study 1

Patients were randomized in a 1:1:1:1 ratio to receive enerisant at 25, 50, and 100 mg or placebo once daily in the morning during treatment period A. For treatment period B, patients in the enerisant 25, 50, and 100 mg groups continued to take the same dose, whereas patients in the placebo group were grouped in a 1:1:1 ratio to receive enerisant at 25, 50, or 100 mg. The doses were set at 25, 50, and 100 mg based on the safety and pharmacokinetic findings in healthy volunteers confirmed in the phase 1 study [Unpublished data]. Participants were randomized by the stratified block randomization method with the presence or absence of cataplexy as the stratification factor. Randomization was performed by an independent company using a computer-generated list of random numbers. Throughout the study, the patients, site investigators, and raters remained blinded. For safety reasons, allocation to the enerisant 100 mg group was terminated in the middle of the study.

#### Study 2

**Fig. 2:**
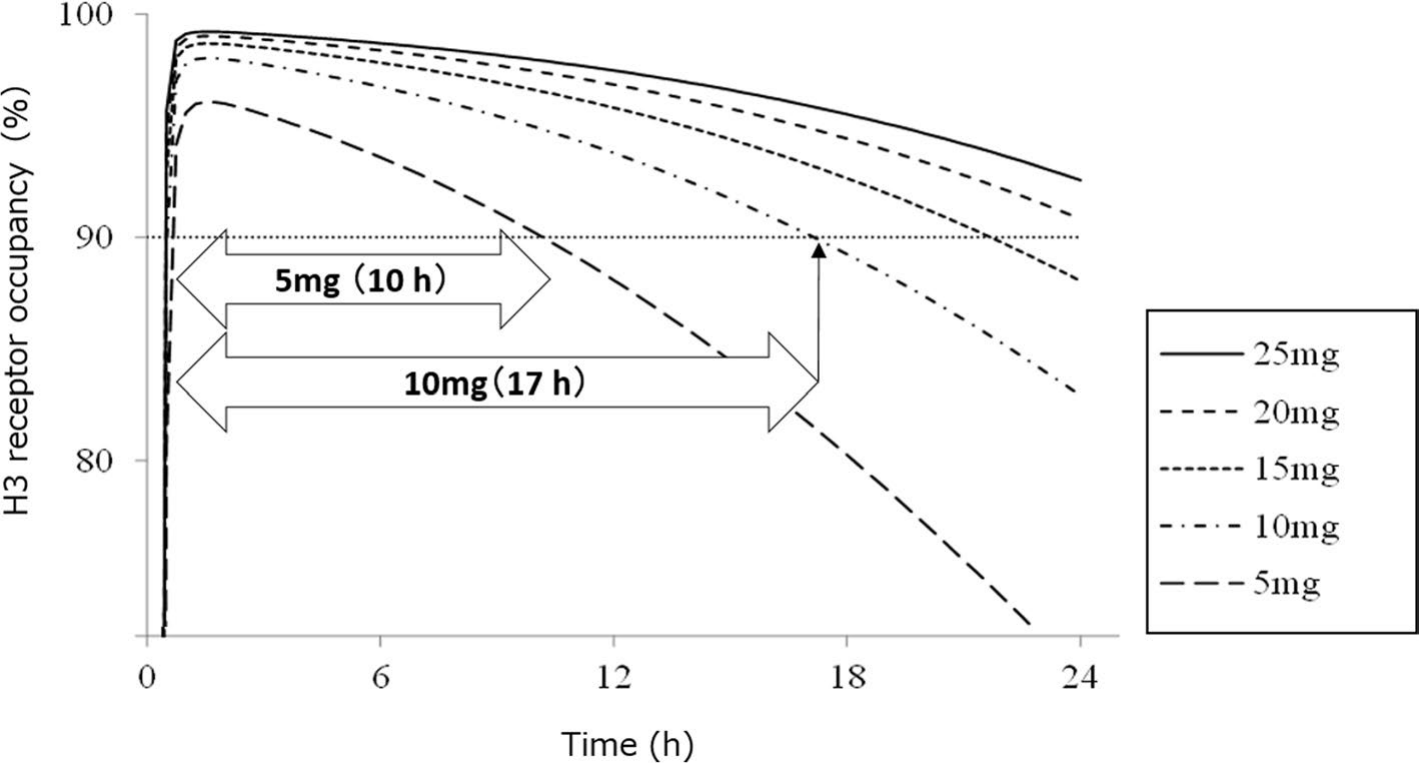
Simulation of H3 receptor occupancy after enerisant administration. The time-course of histamine H3 receptor occupancy after a single oral administration of enerisant was simulated, and the time to maintain 90% of H3 receptor occupancy was calculated. In the simulation, pharmacokinetic data obtained from the enerisant single ascending dose study and the pharmacodynamics data obtained from the H3 receptor occupancy study of enerisant [9] were used. Phoenix WinNonlin (ver.6.2) and Microsoft Excel 2010 were used as the analysis software

Patients were randomized in a 1:1:1 ratio to receive enerisant at 5 and 10 mg or placebo once daily in the morning. Participants were randomized by the stratified block randomization method with the country of origin as the stratification factor. Randomization was performed by an independent company using a computer-generated list of random numbers. Throughout the study, the patients, site investigators, and raters remained blinded. Adequate efficacy can be expected at 5 and 10 mg, according to the results of the intracerebral histamine H3 receptor occupancy study of healthy individuals [9] (Fig. 2). Based on a PET study using Rhesus monkeys, it was expected that > 90% occupancy of the H3 receptor by enerisant promotes arousal. This is consistent with a previous report showing that up to 90% of human H3 receptor occupancy by MK-7288 has shown efficacy for EDS due to obstructive sleep apnea [15]. The duration of H3 receptor occupancy at a rate of 90% was expected to range between 10 and 17 h with enerisant 5 and 10 mg administration, respectively. Thus, the doses were expected to maintain daytime wakefulness without night-time insomnia.

### Outcomes

#### Efficacy

The efficacy outcomes included the objective and subjective measures of EDS. The primary endpoint for efficacy was a change from baseline to week 3 (the end of period A) in the mean SL of the four MWT sessions in Study 1 and Study 2, respectively. MWT consisted of four 20-min periods with a 2-h interval among respective sessions. This 20-min protocol was the standard method used in multiple previous trials, ensuring an objective assessment of sleepiness in patients with narcolepsy [16]. SL was defined as the time from lights out to the first three consecutive epochs of stage N1 or one epoch of any other stage of sleep as measured by electroencephalogram [17].

The secondary endpoints included changes from the baseline to the end of the study in the Epworth Sleepiness Scale (ESS), a patient-reported measure of EDS, change from baseline to the end of the study in SL of each MWT session, sleep parameters on overnight PSG at the end of the study, and the weekly number of episodes of narcolepsy-related symptoms (i.e., sleep paralysis and cataplexy) reported in patients’ sleep diaries.

#### Safety

The following safety endpoints were assessed: adverse events (AEs), body weight, laboratory tests, vital signs, 12-lead electrocardiogram (ECG), suicidality evaluated using Columbia-Suicide Severity Rating Scale (C-SSRS), and dependency evaluated using a dependency questionnaire.

### Statistics

For both studies, the full analysis set (FAS) was used as the primary analysis set. Regarding the secondary analysis set, the per protocol set (PPS) was used for the efficacy evaluation, whereas the safety analysis set (SAS) was used for the safety evaluation.

The FAS included patients who received at least one dose of the study drug and had at least one set of efficacy data. The PPS included patients without any serious protocol violations who had available primary endpoint data. The SAS included patients who received at least one dose of the study drug.

Patients’ demographics and other baseline characteristics (MWT and ESS) were summarized using descriptive statistics. Primary and secondary efficacy endpoint data were presented as mean values and standard deviations. The least-squares mean differences were compared with the placebo, and the associated 95% confidence intervals were calculated using analysis of covariance with the baseline as the covariate in Study 1 and using analysis of covariance with the baseline and country (Japan or Korea) as covariates in Study 2. Missing data were not imputed, and data were not adjusted for multiplicity. The two-sided significance level was set at 5%. Statistical analyses were performed using SAS (SAS Institute, Tokyo, Japan) version 9.2 in Study 1 and version 9.4 in Study 2.

## Results

### Study population and characteristics

#### Study 1

**Fig. 3:**
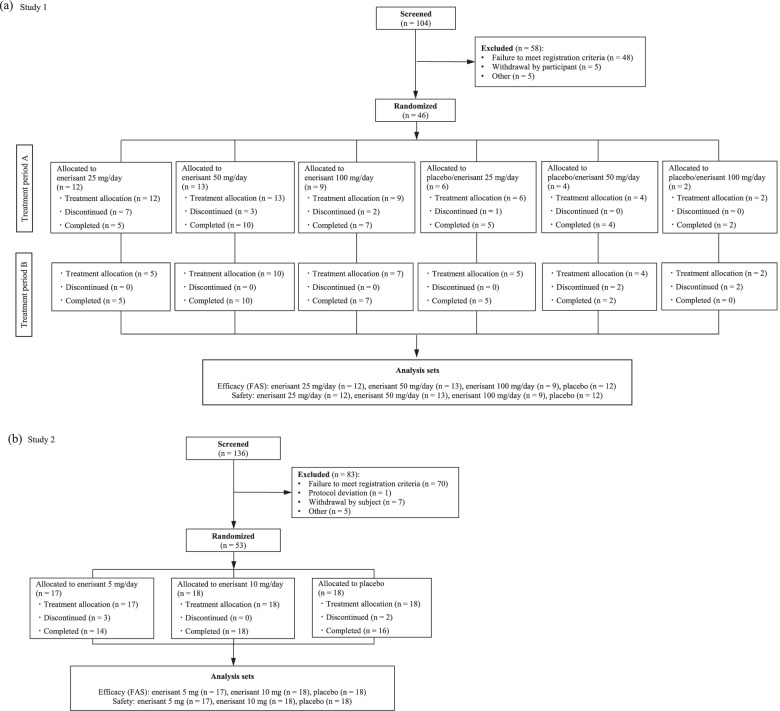
Disposition of subjects for Study 1 (**a**) and Study 2 (**b**)

Among the 104 study candidates (range for patient recruitment and follow-up: June 14, 2014 to April 6, 2016), 46 were randomized and allocated to the investigational product group (Fig. 3a). The most common reason for screening failures was not meeting eligibility criteria (*n* = 48). Among the 46 participants who received the investigational product (FAS), 13 withdrew during treatment period A due to AEs (7 participants in the 25 mg group, 3 in the 50 mg group, and 2 in the 100 mg group) and other reason (1 participant in the placebo group). Among the 33 patients who completed treatment period A, 4 withdrew during treatment period B due to AEs (2 participants in the placebo/enerisant 50 mg group, and 2 in the placebo/enerisant 100 mg group).

Demographics and the other baseline characteristics of the FAS are summarized by treatment groups (Table 1). No statistically significant difference was observed in any baseline characteristics among the treatment groups. The mean age was 27.2 ± 5.5 years, body weight was 63.07 ± 11.70 kg, body mass index was 23.28 ± 3.64 kg/m^2^, and duration of disease was 1.82 ± 2.71 years. The mean SL on MWT was 2.63 ± 2.00 min, and the ESS score was 18.4 ± 3.2, without apparent differences between the groups.

**Table 1:** Demographics and other baseline characteristics in Study 1 and Study 2 (FAS)

Variables	Study 1					Study 2				
	Enerisant 25 mg	Enerisant 50 mg	Enerisant 100 mg	Placebo	Total		Enerisant 5 mg	Enerisant 10 mg	Placebo	Total
	(*n* = 12)	(*n* = 13)	(*n* = 9)	(*n* = 12)	(*n* = 46)		(*n* = 17)	(*n* = 18)	(*n* = 18)	(*n* = 53)
Age, y, mean ± SD	28.9 ± 5.7	26.1 ± 5.6	28.6 ± 4.3	25.8 ± 5.9	27.2 ± 5.5		26.9 ± 9.7	26.2 ± 8.0	29.4 ± 10.4	27.5 ± 9.3
Sex, n (%)										
Female	4 (33.3)	8 (61.5)	6 (66.7)	5 (41.7)	23 (50.0)		9 (52.9)	10 (55.6)	8 (44.4)	27 (50.9)
Male	8 (66.7)	5 (38.5)	3 (33.3)	7 (58.3)	23 (50.0)		8 (47.1)	8 (44.4)	10 (55.6)	26 (49.1)
Country, n (%)										
Japan	-	-	-	-	-		13 (76.5)	13 (72.2)	14 (77.8)	40 (75.5)
Korea	-	-	-	-	-		4 (23.5)	5 (27.8)	4 (22.2)	13 (24.5)
Body weight (kg, mean ± SD)	62.76 ± 8.19	59.97 ± 11.66	62.58 ± 14.33	67.11 ± 12.86	63.07 ± 11.70		66.01 ± 14.83	62.19 ± 14.53	62.77 ± 13.91	63.61 ± 14.24
Body mass index (kg/m^2^, mean ± SD)	22.83 ± 2.30	22.38 ± 2.90	23.80 ± 4.85	24.32 ± 4.50	23.28 ± 3.64		24.22 ± 4.92	23.31 ± 4.39	23.16 ± 4.37	23.55 ± 4.49
Diagnosis, n (%)										
Narcolepsy with cataplexy	6 (50.0)	7 (53.8)	6 (66.7)	7 (58.3)	26 (56.5)		-	-	-	-
Narcolepsy without cataplexy	6 (50.0)	6 (46.2)	3 (33.3)	5 (41.7)	20 (43.5)		-	-	-	-
Narcolepsy type 1	-	-	-	-	-		5 (29.4)	9 (50.0)	6 (33.3)	20 (37.7)
Narcolepsy type 2	-	-	-	-	-		12 (70.6)	9 (50.5)	12 (66.7)	33 (62.3)
Duration of disease (year, mean ± SD)	1.33 ± 2.30	1.42 ± 1.80	3.28 ± 4.06	1.64 ± 2.65	1.82 ± 2.71		2.08 ± 1.82	2.28 ± 3.37	1.85 ± 2.33	2.08 ± 2.56
Mean SL in baseline MWT, (min, mean ± SD)	2.12 ± 1.77	3.22 ± 2.45	2.46 ± 1.54	2.64 ± 2.06	2.63 ± 2.00		5.40 ± 3.04	5.08 ± 2.49	4.56 ± 3.24	5.01 ± 2.90
Baseline total ESS score, (mean ± SD)	19.3 ± 2.5	19.4 ± 2.6	18.0 ± 3.4	16.7 ± 4.0	18.4 ± 3.2		16.8 ± 3.6	17.8 ± 3.8	17.2 ± 2.8	17.3 ± 3.4

*SD* standard deviation, *SL* sleep latency, *MWT* maintenance of wakefulness test, and *ESS* Epworth Sleepiness Scale

#### Study 2

Of the 136 study candidates (range for patient recruitment and follow-up: October 31, 2017 to December 13, 2018), 53 were randomized and allocated to the investigational product group (Fig. 3b). The most common reason for screening failures was not meeting eligibility criteria (*n* = 70). Among the 53 participants who received the investigational product (FAS), 5 withdrew from the study due to AEs (3 participants in the enerisant 5 mg group and 1 in the placebo group) and other reason (1 participant in the placebo group).

Demographic and other baseline characteristics for FAS are summarized for respective treatment groups (Table 1). No statistically significant difference among treatment groups was observed in any baseline variables. The mean age was 27.5 ± 9.3 years, body weight was 63.61 ± 14.24 kg, body mass index was 23.55 ± 4.49 kg/m^2^, and duration of disease was 2.08 ± 2.56 years. The percentage of patients diagnosed with narcolepsy type 1 was 37.7% and that of patients with narcolepsy type 2 was 62.3%. Mean SL on MWT was 5.01 ± 2.90 min, and the ESS score was 17.3 ± 3.4, without apparent differences between the groups.

### Efficacy

#### Study 1

Regarding the primary endpoint, the change in mean SL on MWT was 0.53 ± 2.75 min in the 25 mg group, 0.33 ± 3.72 min in the 50 mg group, − 0.16 ± 2.73 min in the 100 mg group, and − 0.88 ± 2.40 min in the placebo group. No statistically significant difference was observed between the placebo and enerisant groups (Table 2). The MWT outcomes in the narcolepsy subgroups with and without cataplexy in Study 1 are provided in Supplementary Table S1.

**Table 2:** Primary endpoint outcomes: MWT changes from baseline in Study 1 and Study 2

Group	Study 1				Study 2		
	Enerisant 25 mg	Enerisant 50 mg	Enerisant 100 mg	Placebo	Enerisant 5 mg	Enerisant 10 mg	Placebo
	(*n* = 4)	(*n* = 10)	(*n* = 7)	(*n* = 11)	(*n* = 14)	(*n* = 18)	(*n* = 16)
SL at baseline (min)	2.12 ± 1.77	3.22 ± 2.45	2.46 ± 1.54	2.64 ± 2.06	5.40 ± 3.04	5.08 ± 2.49	4.56 ± 3.24
SL at week 3 (min)	3.50 ± 4.69	3.80 ± 4.52	2.53 ± 1.85	1.65 ± 1.98	5.43 ± 4.41	5.74 ± 4.73	4.84 ± 5.20
Change from baseline to week 3 (min)	0.53 ± 2.75	0.33 ± 3.72	− 0.16 ± 2.73	− 0.88 ± 2.40	0.73 ± 3.42	0.66 ± 3.44	0.22 ± 4.90
Difference compared with placebo [95% CI]	1.54 [− 2.00, − 5.08]	1.50 [− 1.19, − 4.18]	0.77 [− 2.16, − 3.70]	-	0.43 [− 2.45, − 3.31]	0.41 [− 2.30, − 3.13]	–
*p* value	0.380	0.263	0.594		0.765	0.761	

mean ± SD

*CI* confidence interval, *SD* standard deviation, *SL* sleep latency, *MWT* maintenance of wakefulness test

**Fig. 4:**
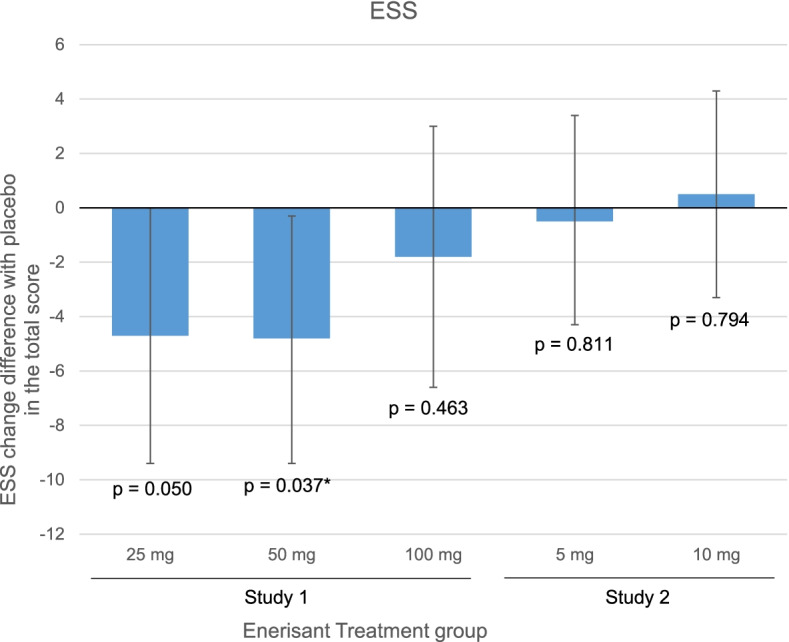
Secondary endpoint outcomes: total ESS score changes in Study 1 and Study 2. 95% confidence interval

With regard to one of the secondary efficacy endpoints, the mean change from the baseline total ESS score was − 8.0 ± 7.0 in the 25 mg group, − 8.3 ± 5.8 in the 50 mg group, − 4.2 ± 6.2 in the 100 mg group, and − 1.5 ± 3.5 in the placebo group (Table 3). The total ESS score decreased from week 1 to the end of treatment period A in the enerisant 50 mg group, and a similar trend was observed in the other enerisant groups. The mean change in the total ESS score was statistically higher in the 50 mg group than in the placebo group (*p* = 0.037) (Fig. 4). Results of the other secondary efficacy endpoint, cataplexy incidence, are provided in Supplementary Table S2.

**Table 3:** Secondary endpoint outcomes: ESS changes from baseline in Study 1 and Study 2

Group	Study 1				Study 2		
	Enerisant 25 mg	Enerisant 50 mg	Enerisant 100 mg	Placebo	Enerisant 5 mg	Enerisant 10 mg	Placebo
	(*n* = 11)	(*n* = 13)	(*n* = 9)	(*n* = 12)	(*n* = 16)	(*n* = 18)	(*n* = 17)
Baseline	19.3 ± 2.5	19.4 ± 2.6	18.0 ± 3.4	16.7 ± 4.0	16.8 ± 3.6	17.8 ± 3.8	17.2 ± 2.8
Week 3^a^	11.2 ± 5.5	11.1 ± 5.4	13.8 ± 4.8	15.2 ± 5.4	12.7 ± 7.6	14.2 ± 4.8	13.2 ± 5.4
Change from baseline to week 3^a^	− 8.0 ± 7.0	− 8.3 ± 5.8	− 4.2 ± 6.2	− 1.5 ± 3.5	− 4.3 ± 6.8	− 3.6 ± 4.6	− 3.9 ± 5.1
Difference compared with placebo [95% CI]	− 4.7 [− 9.4, − 0.0]	− 4.8 [− 9.4, − − 0.3]	− 1.8 [− 6.6, − 3.0]	–	− 0.5 [− 4.3, − 3.4]	0.5 [− 3.3, − 4.3]	–
*p* value	0.050	0.037*	0.463		0.811	0.794	

mean ± SD

*SD* standard deviation, *CI* confidence interval, *ESS*, Epworth Sleepiness Scale

^a^Includes data from subjects who discontinued before the completion of 3 weeks of dosing

#### Study 2

For the primary efficacy endpoint, the change in mean SL on MWT was 0.73 ± 3.42 min in the 5 mg group, 0.66 ± 3.44 min in the 10 mg group, and 0.22 ± 4.90 min in the placebo group (Table 2). The MWT outcomes in patients with narcolepsy type 1 and type 2 in Study 2 are provided in Supplementary Table S2. As a secondary efficacy endpoint, the mean change in the total ESS score was − 4.3 ± 6.8 in the 5 mg group, − 3.6 ± 4.6 in the 10 mg group, and − 3.9 ± 5.1 in the placebo group (Table 3, Fig. 4). In the 5 mg and 10 mg groups, there was no statistically significant difference compared with the placebo group in either mean SL on MWT or ESS score. Results of the other secondary efficacy endpoint, cataplexy incidence, are provided in Supplemental Table S2.

### Safety

#### Study 1

In treatment period A, AEs were reported in 83.3% (10/12), 69.2% (9/13), 100.0% (9/9), and 41.7% (5/12) of the participants in the 25 mg, 50 mg, 100 mg, and placebo groups, respectively (Table 4). There was a significant difference in the incidence rate of AEs among the treatment groups (*p* = 0.021). The most common AEs were insomnia-related in all the enerisant groups. In treatment period A, insomnia-related AEs were reported by 58.3% (7/12), 30.8% (4/13), 88.9% (8/9), and 8.3% (1/12) of the participants in the 25 mg, 50 mg, 100 mg, and placebo groups, respectively. Other common AEs (those reported by at least 2 patients in any treatment group) were headache [25.0% (3/12) in the 25 mg group, 30.8% (4/13) in the 50 mg group, 22.2% (2/9) in the 100 mg group, and 0% (0/12) in the placebo group] and nausea [25.0% (3/12) in the 25 mg group, 7.7% (1/13) in the 50 mg group, 22.2% (2/9) in the 100 mg group, and 0% (0/12) in the placebo group]. There was no dose-dependent increase in the incidence of these AEs. AEs leading to discontinuation in treatment period A were reported by 58.3% (7/12), 23.1% (3/13), 22.2% (2/9), and 0% (0/12) of the participants in the 25 mg, 50 mg, 100 mg, and placebo groups, respectively. From these results, it was speculated that there was a tolerability problem with enerisant. In particular, independent data monitoring of the first 23 patients revealed that many of the participants showing insomnia-related AEs were in the 100 mg group. Given this, we decided not to continue allocation to the 100 mg group. However, AEs leading to death and other severe AEs were not reported in this study, and all of the AEs were mild to moderate in severity (data not shown). Additionally, in other safety variables (laboratory test values, vital signs, 12-lead ECG, and C-SSRS), there were no clinically significant abnormalities or notable trends after dosing.

**Table 4:** Adverse events in Study 1 and Study 2

Group	Study 1				Study 2		
	Enerisant 25 mg	Enerisant 50 mg	Enerisant 100 mg	Placebo	Enerisant 5 mg	Enerisant 10 mg	Placebo
	(*n* = 12)	(*n* = 13)	(*n* = 9)	(*n* = 12)	(*n* = 17)	(*n* = 18)	(*n* h 18)
Any AE	10 (83.3)	9 (69.2)	9 (100.0)	5 (41.7)	12 (70.6)	9 (50.0)	5 (27.8)
Serious AEs	0	0	0	0	0	0	0
Discontinuations due to AEs	7 (58.3)	3 (23.1)	2 (22.2)	0	3 (17.6)	0	1 (5.6)
Most common AEs (> 10% of patients in any group)							
Headache	3 (25.0)	4 (30.8)	2 (22.2)	0	4 (23.5)	3 (16.7)	1 (5.6)
Nausea	3 (25.0)	1 (7.7)	2 (22.2)	0	2 (11.8)	2 (11.1)	2 (11.1)
Insomnia^a^	7 (58.3)	4 (30.8)	8 (88.9)	1 (8.3)	4 (23.5)	3 (16.7)	0
Dizziness	0	1 (7.7)	0	0	3 (17.6)	0	0
Dysmenorrhea	0	0	0	0	0	2 (11.1)	0
Palpitations	1 (8.3)	2 (15.4)	0	1 (8.3)	0	0	0
Nasopharyngitis	0	0	0	2 (16.7)	0	1 (5.6)	0
Parosmia	2 (16.7)	0	0	0	0	0	0

^a^MedDRA PT: insomnia, initial insomnia, and middle insomnia

*AE* adverse event

#### Study 2

AEs were reported by 70.6% (12/17), 50.0% (9/18), and 27.8% (5/18) of the participants in the 5 mg, 10 mg, and placebo groups, respectively (Table 4). The most common AEs (those reported by at least 2 participants in any treatment group) were insomnia-related (4 participants in the 5 mg group and 3 in the 10 mg group), headache (4 participants in the 5 mg group, 3 in the 10 mg group, and 1 in the placebo group), nausea (2 participants each in the 5 mg, 10 mg, and placebo groups), dizziness (3 participants in the 5 mg group), and dysmenorrhea (2 participants in the 10 mg group). AEs leading to discontinuation of the study drugs were reported by 17.6% (3/17) and 5.6% (1/18) of the participants in the 5 mg and placebo groups, respectively. AEs leading to death and other severe AEs were not reported. All AEs were mild to moderate in severity (data not shown). Additionally, in other safety variables (laboratory test values, vital signs, 12-lead ECG, and C-SSRS), there were no clinically significant abnormalities or notable trends after dosing. Overall, enerisant at doses of 5 and 10 mg was well tolerated, and its safety was confirmed.

## Discussion

Two phase 2 dose-finding studies were conducted to evaluate the efficacy and safety of enerisant in patients with narcolepsy. The efficacy of enerisant was partially confirmed in Study 1, which used higher doses (25, 50, and 100 mg/day), but could not be confirmed in Study 2, which used lower doses (5 and 10 mg/day). The higher doses were not sufficiently tolerated, and there were many withdrawals due to AEs (mainly insomnia, headache, and nausea). In contrast, in Study 2, both doses (5 and 10 mg/day) were well tolerated, with a lower incidence of AEs reported in Study 2 than in Study 1.

In Study 1, the highest dose was set at 100 mg, because it was tolerated in a previous MAD study conducted on healthy adults, and the lowest dose was set at 25 mg, as the estimated potential effective dose from the results of nonclinical pharmacology studies conducted on cynomolgus monkeys [unpublished data]. Contrary to the results of the MAD study, none of the 25–100 mg/day doses were sufficiently tolerated in patients with narcolepsy. The AEs were similar between the MAD study and Study 1, and insomnia was the most frequently reported AE. However, the frequency of insomnia and the rate of withdrawal due to the occurrence of AEs (overall) were much higher in Study 1 than in the MAD study.

Although the precise reason for the higher frequency of insomnia with enerisant treatment in patients with narcolepsy than in healthy subjects is unknown, it may be ascribed to a difference in the activities of histaminergic neurons. Indeed, it has been reported that patients with narcolepsy had a 94% greater number of tuberomammillary nucleus histaminergic neurons compared with non-narcoleptic controls [18]. Given this, it is conceivable that sensitivity to H3 receptor antagonists may differ between patients with narcolepsy and healthy adults, possibly leading to the difference in tolerability to enerisant.

In Study 1, the changes in the ESS score among all the enerisant groups seemed to be larger, and a significant difference compared with the placebo group was seen in the 50 mg/day group, suggesting that enerisant has the potential to improve daytime sleepiness in patients with narcolepsy. On the other hand, the changes in mean SL on MWT in the enerisant groups were small and did not show statistical difference compared with those in the placebo group. Discrepancies between these two endpoints have been shown in many reports [19, 20]. MWT is an objective index of the inability to maintain wakefulness, whereas ESS is a subjective self-reported index of sleepiness [19]. Thus, differences in the characteristics of these measures might have contributed to the discrepancy in the results. ESS was authorized as a primary endpoint in the EU pitolisant study [21]; thus, ESS could be reliable to evaluate daytime sleepiness in clinical trials with H3 receptor antagonists. Notably, the average baseline MWT in Study 1 was 2.6 min, which is lower than the previously reported baseline SLs on MWT in the modafinil and pitolisant clinical trials [21, 22]. Therefore, it is possible that patients with more severe symptoms were recruited in Study 1 than in the previously conducted trials, possibly hampering efficacy detection of enerisant due to the “floor effect.”

When we planned Study 2, the doses were set at a much lower level based on the low tolerability in Study 1, and patients with relatively larger SLs on MWT were included, taking the floor effect into consideration. In particular, we simulated the doses in Study 2 based on the time-course of cerebral H3 receptor occupancy of enerisant in healthy adults obtained in a PET study [9]. The receptor occupancy simulation employed in Study 2 indicated that the dose of 5 mg occupies H3 receptor by more than 90% for 10 h after administration. Likewise, the maximum dose was set at 10 mg, which occupies H3 receptor by more than 90% for 17 h. Thus, 5 mg was speculated not to induce insomnia theoretically, and AEs, including insomnia, were expected to be markedly reduced at 10 mg. According to our simulation, all doses used in Study 1 were estimated to occupy H3 receptor by more than 90% for 24 h, which might have led to the high incidence of insomnia, even at the lowest dose (25 mg/day).

As expected, there was a reduction in the frequency of AEs (including insomnia) and fewer withdrawals due to AEs in Study 2 than in Study 1, despite the small number of patients in both the 5 mg and 10 mg groups with insomnia-related AEs. On the other hand, although some patients showed some improvement in MWT or ESS, the efficacy expected from the receptor occupancy simulation was not observed in either of these two measures in Study 2. The variations in the efficacy and safety of enerisant among patients might indicate the existence of individual sensitivity to this drug. Given this, both efficacy and tolerability could not be fully predicted only from the receptor occupancy simulation of enerisant, and individual differences in patients need to be considered.

As enerisant is rarely metabolized by CYP, individual differences in its plasma concentration have been speculated to be relatively low [8]. Therefore, we initially speculated that the optimal fixed dose for patients with narcolepsy would be in the range of 5–100 mg. However, contrary to our hypothesis, the efficacy and safety were significantly different among patients, including differences in tolerability between healthy adults and patients with narcolepsy. As such, the optimal dose could not be determined in the fixed-dose studies.

As for pitolisant, the approved dose ranges widely from 4.5 to 36 mg, and patient-specific dose adjustment is recommended considering the efficacy, safety, and influences of drugs metabolized by CYPs [23]. Therefore, interindividual variabilities in terms of efficacy and safety may be common between H3 receptor antagonists, independent of their pharmacological or pharmacokinetic profiles. In addition, doses of pitolisant up to 36 mg, taken once daily, show efficacy and are tolerated [24]. In our study, sufficient efficacy and tolerability at higher doses of enerisant were observed in some patients, but any firm conclusions on this aspect cannot be drawn in this study. The differences in the results between pitolisant and enerisant possibly stem from the differences in the study design (e.g., dose-titration design vs. fixed-dose design).

The detailed mechanisms of histamine action remain unclear; however, it is noteworthy that even enerisant, which has a better PK profile than pitolisant, showed large individual differences in efficacy and tolerability. In addition to differences in sensitivity to AEs between healthy adults and patients with narcolepsy, changes in histaminergic neuronal activities may differ even among patients with narcolepsy, both qualitatively and quantitatively. This may explain the large individual differences in the efficacy and safety of H3 receptor antagonists. Because H3 receptor antagonists exert their effect by increasing histamine release and subsequent stimulation of postsynaptic H1 receptor, the determination of H1 receptor occupancy with released histamine by H3 receptor antagonists would help clarify this issue.

As the first limitation, the present study possibly had sampling bias coming from the additional inclusion/exclusion criteria items (i.e. the weekly frequency of daytime dozing off or SL on MWT at the baseline) for precisely evaluating the effect of study drugs. As the second limitation, especially in Study 1 many participants discontinued treatment due to AEs, and the limited number of patients who underwent post-treatment MWT made it difficult to interpret the results on efficacy.

## Conclusions

In the present two studies, it was not possible to determine the optimal dose of enerisant for the treatment of EDS in patients with narcolepsy. As enerisant showed large interindividual variability in its efficacy and safety despite its favorable PK profile, tailored dosage adjustments based on individual efficacy and safety would be required. We hope that future studies reveal the efficacy and safety of enerisant for the treatment of narcolepsy and other neuropsychiatric disorders for which H3 receptor antagonists can be indicated.

## Supplementary Information

**Additional file 1: Table S1.** The MWT outcomes in the narcolepsy subgroups with and without cataplexy in Study 1. **Table S2.** Weekly incidence of cataplectic episodes in Study 1 and Study 2. **Table S3.** The MWT outcomes in patients with narcolepsy type 1 and type 2 in Study 2.

**Additional file 2.** List of the ethics committees.

## Data Availability

The data that support the findings of this study are available from Taisho Pharmaceutical Co., Ltd. However, restrictions apply to the availability of these data due to their use under license for the current study as they are not currently publicly available. Data are however available from the authors upon reasonable request and with the permission of Taisho Pharmaceutical Co., Ltd.

## Abbreviations

AASM: American Academy of Sleep Medicine
AE: Adverse event
C-SSRS: Columbia-Suicide Severity Rating Scale
CYP: Cytochrome P450
DDI: Drug–drug interaction
ECG: Electrocardiogram
EDS: Excessive daytime sleepiness
ESS: Epworth Sleepiness Scale
FAS: Full analysis set
ICSD-2: The second edition of the International Classification of Sleep Disorders
ICSD-3: The third edition of the International Classification of Sleep Disorders
MAD: Multiple ascending dose
MWT: Maintenance of wakefulness test
PET: Positron emission tomography
PK: Pharmacokinetic
PPS: Per protocol set
PSG: Polysomnography
RO: Receptor occupancy
SAD: Single ascending dose
SAS: Safety analysis set
SD: Standard deviation
SL: Sleep latency
TST: Total sleep time
WASO: Wake time after sleep onset
