# Supplementary material for: Optimal dose determination of enerisant (TS-091) for patients with narcolepsy: two randomized, double-blind, placebo-controlled trials
Source: BMC Psychiatry. 2022 Feb 22;22:141. doi: 10.1186/s12888-022-03785-7 (PMC8862520; doi:10.1186/s12888-022-03785-7)
Supplement: Supplementary file 1 — Additional file 1: Table S1. The MWT outcomes in the narcolepsy subgroups with and without cataplexy in Study 1. Table S2. Weekly incidence of cataplectic episodes in Study 1 and Study 2. Table S3. The MWT outcomes in patients with narcolepsy type 1 and type 2 in Study 2. [file 12888_2022_3785_MOESM1_ESM.docx]

**Supplemental Table S1. The MWT outcomes in the narcolepsy subgroups with and without cataplexy in Study 1.**

| Study 1 | Group | Enerisant | Enerisant | Enerisant | Placebo |
| --- | --- | --- | --- | --- | --- |
|  |  | 25 mg | 50 mg | 100 mg |  |
| Narcolepsy | N | 0 | 4 | 5 | 7 |
| with cataplexy | Baseline | NC | 2.43 ± 1.73 | 2.60 ± 2.03 | 1.99 ± 2.26 |
|  | Week 3^a^ | NC | 3.15 ± 4.42 | 1.86 ± 0.99 | 0.86 ± 0.80 |
|  | Change from | NC | 0.73 ± 2.70 | –0.74 ± 2.45 | –1.13 ± 2.21 |
|  | baseline to week 3^a^ |  |  |  |  |
| Narcolepsy | N | 4 | 6 | 2 | 4 |
| without cataplexy | Baseline | 2.98 ± 2.75 | 4.17 ± 3.24 | 2.90 ± 0.85 | 3.50 ± 1.70 |
|  | Week 3^a^ | 3.50 ± 4.69 | 4.23 ± 4.95 | 4.20 ± 2.97 | 3.05 ± 2.77 |
|  | Change from | 0.53 ± 2.75 | 0.07 ± 4.51 | 1.30 ± 3.82 | –0.45 ± 3.01 |
|  | baseline to week 3^a^ |  |  |  |  |
| mean ± SD, min | | | | | |
| SD: standard deviation; NC: not calculated | | | | | |
| a, Includes data from subjects who discontinued before the completion of 3 weeks of dosing | | | | | |

**Supplemental Table S2. Weekly incidence of cataplectic episodes in Study 1 and Study 2.**

| Group | Study 1 | | | | Study 2 | | |
| --- | --- | --- | --- | --- | --- | --- | --- |
|  | Enerisant 25 mg | Enerisant 50 mg | Enerisant 100 mg | Placebo | Enerisant 5 mg | Enerisant 10 mg | Placebo |
|  | (n = 11) | (n = 13) | (n = 9) | (n = 12) | (n = 17) | (n = 18) | (n = 18) |
| Baseline | 3.3 ± 7.5 | 2.4 ± 7.1 | 0.2 ± 0.4 | 5.6 ± 11.2 | 1.2 ± 3.7 | 1.3 ± 2.5 | 1.0 ± 3.0 |
| Week 3^a^ | 1.4 ± 2.7 | 0.5 ± 1.2 | 0.6 ± 1.7 | 3.8 ± 7.0 | 0.6 ± 2.4 | 0.6 ± 1.2 | 1.1 ± 3.3 |
| Change from  baseline to week 3^a^ | –2.3 ± 5.3 | –1.9 ± 6.1 | 0.3 ± 1.4 | –1.8 ± 5.2 | –0.5 ± 3.8 | –0.7 ± 1.6 | 0.1 ± 0.5 |
| Difference compared with  placebo [95% CI] | –1.6 [–3.4,–0.2] | –1.9 [–3.7,– –0.2] | –0.9 [–2.9,–1.0] | – | –0.5 [–1.8,–0.8] | –0.6 [–1.9,–0.6] | – |
| p value | 0.089 | 0.031* | 0.353 |  | 0.436 | 0.313 |  |
| mean ± SD, times/week | | | | | | | |
| SD: standard deviation; CI: confidence interval | | | | | | | |
| a, Includes data from subjects who discontinued before the completion of 3 weeks of dosing | | | | | | | |

**Supplemental Table S3. The MWT outcomes in patients with narcolepsy type 1 and type 2 in Study 2**

| Study 2 | Group | Enerisant | Enerisant | Placebo |
| --- | --- | --- | --- | --- |
|  |  | 5 mg | 10 mg |  |
| Narcolepsy | N | 4 | 9 | 6 |
| Type 1 | Baseline | 4.13 ± 3.03 | 4.19 ± 1.48 | 5.20 ± 3.53 |
|  | Week 3^a^ | 2.18 ± 1.02 | 4.70 ± 4.29 | 3.95 ± 3.59 |
|  | Change from | –1.95 ± 2.13 | 0.51 ± 3.36 | –1.25 ± 2.35 |
|  | baseline to week 3^a^ |  |  |  |
| Narcolepsy | N | 10 | 9 | 10 |
| Type 2 | Baseline | 4.93 ± 2.77 | 5.98 ± 3.03 | 4.28 ± 3.52 |
|  | Week 3^a^ | 6.73 ± 4.60 | 6.78 ± 5.17 | 5.38 ± 6.09 |
|  | Change from | 1.80 ± 3.31 | 0.80 ± 3.72 | 1.10 ± 5.89 |
|  | baseline to week 3^a^ |  |  |  |
| mean ± SD, min | | | | |
| SD: standard deviation | | | | |
| a, Includes data from subjects who discontinued before the completion of 3 weeks of dosing | | | | |
