# Supplementary material for: Optimal dose determination of enerisant (TS-091) for patients with narcolepsy: two randomized, double-blind, placebo-controlled trials
Source: BMC Psychiatry. 2022 Feb 22;22:141. doi: 10.1186/s12888-022-03785-7 (PMC8862520; doi:10.1186/s12888-022-03785-7)
Supplement: Supplementary file 2 — Additional file 2. List of the ethics committees. [file 12888_2022_3785_MOESM2_ESM.docx]

**List of the ethics committees**

**Study 1**

| No. | Site | Institutional review board |
| --- | --- | --- |
| 1 | Akita University Hospital | Akita University Hospital IRB |
| 2 | Otsuki Sleep Clinic | Aida Hospital IRB |
| 3 | National Center of Neurology and Psychiatry | National Center of Neurology and Psychiatry IRB |
| 4 | Yoyogi Sleep Disorder Center | Institute of Neuropsychiatry IRB |
| 5 | Gifu Mates Sleep Clinic | Toyohashi Mates Sleep Treatment Clinic IRB |
| 6 | Iwata Mates Sleep Disorders Treatment Clinic | Toyohashi Mates Sleep Treatment Clinic IRB |
| 7 | Toyohashi Mates Sleep Treatment Clinic | Toyohashi Mates Sleep Treatment Clinic IRB |
| 8 | Osaka Kaisei Hospital | Osaka Kaisei Hospital IRB |
| 9 | Hannan Hospital | Hannan Hospital IRB |
| 10 | Ehime University Hospital | Ehime University Hospital IRB |
| 11 | Kochi Kagamigawa Hospital | Joint Institutional Review Board |
| 12 | You Ariyoshi Sleep Clinic | Toyohashi Mates Sleep Treatment Clinic IRB |
| 13 | Fukuoka Urasoe Clinic | Joint Institutional Review Board |
| 14 | Kurume University Hospital | Kurume University Hospital IRB |
| 15 | Kirigaoka Tsuda Hospital | Kirigaoka Tsuda Hospital IRB |
| 16 | Inoue Hospital | Nihonbashi Sakura Clinic IRB |
| 17 | Kuwamizu Hospital | Kuwamizu Hospital IRB |
| 18 | Junwakai Memorial Hospital | Junwakai Memorial Hospital IRB |
| 19 | Nakamura Clinic | Urasoe General Hospital IRB |

**Study 2**

| No. | Site | Institutional review board |
| --- | --- | --- |
| 1 | Otsuki Sleep Clinic | Aida Hospital IRB |
| 2 | Hirasawa Sleep Mental Clinic | Yokohama Minoru Clinic IRB |
| 3 | Good-Sleep Clinic | Nihonbashi Sakura Clinic IRB |
| 4 | Yoyogi Sleep Disorder Center | Institute of Neuropsychiatry IRB |
| 5 | Good-Sleep Omori Clinic | Nihonbashi Sakura Clinic IRB |
| 6 | Ota Memorial Center | Yokohama Minoru Clinic IRB |
| 7 | Takaoka Clinic | Kitamachi Clinic IRB |
| 8 | Aichi Medical University Hospital | Aichi Medical University Hospital IRB |
| 9 | Tanaka Sleep Clinic | Jimbo Orthopedics IRB |
| 10 | Osaka Kaisei Hospital | Osaka Kaisei Hospital IRB |
| 11 | Hannan Hospital | Hannan Hospital IRB |
| 12 | Kyotani Clinic | Tomisaka Clinic IRB |
| 13 | Ehime University Hospital | Ehime University Hospital IRB |
| 14 | You Ariyoshi Sleep Clinic | Yokohama Minoru Clinic IRB |
| 15 | Fukuoka Urasoe Clinic | Yokohama Minoru Clinic IRB |
| 16 | Kurume University Hospital | Yokohama Minoru Clinic IRB |
| 17 | Inoue Hospital | Kitamachi Clinic IRB |
| 18 | Kotorii Isahaya Hospital | Haradoi Hospital IRB |
| 19 | Kuwamizu Hospital | Kuwamizu Hospital IRB |
| 20 | The Catholic University of Korea St. Vincent Hospital | The Catholic University of Korea St. Vincent Hospital IRB |
| 21 | Samsung Medical Center | Samsung Medical Center IRB |
| 22 | Seoul National University Hospital | Seoul National University Hospital IRB |
| 23 | KyungHee University at Gangdong | KyungHee University at Gangdong IRB |
